# Repeated home drinking water sampling to improve detection of particulate lead spikes: a simulation study

**DOI:** 10.1038/s41370-023-00534-0

**Published:** 2023-04-03

**Authors:** Samuel Dorevitch, Sarah D. Geiger, Walton Kelly, David E. Jacobs, Hakan Demirtas

**Affiliations:** 1 Division of Environmental and Occupational Health Sciences, University of Illinois Chicago School of Public Health, Chicago, IL USA; 2 Institute for Environmental Science and Policy, University of Illinois Chicago, Chicago, IL USA; 3 Department of Kinesiology and Community Health, University of Illinois Urbana-Champaign, Urbana, IL USA; 4 University of Illinois Urbana-Champaign, Prairie Research Institute, Illinois State Water Survey, Champaign, IL USA; 5 National Center for Healthy Housing, Columbia, MD USA; 6 Division of Epidemiology and Biostatistics, University of Illinois Chicago School of Public Health, Chicago, IL USA

**Keywords:** Drinkingwater, Lead, Environmental monitoring, Lead and Copper Rule, Lead poisoning prevention.

## Abstract

**Background:**

Lead can be present in drinking water in soluble and particulate forms. The intermittent release of lead particulates in drinking water can produce highly variable water lead levels (WLLs) in individual homes, a health concern because both particulate and soluble lead are bioavailable. More frequent water sampling would increase the likelihood of identifying sporadic lead “spikes,” though little information is available to aid in estimating how many samples are needed to achieve a given degree of sensitivity to spike detection.

**Objective:**

To estimate the number of rounds of tap water sampling needed to determine with a given level of confidence that an individual household is at low risk for the intermittent release of lead particulates.

**Methods:**

We simulated WLLs for 100,000 homes on 15 rounds of sampling under a variety of assumptions about lead spike release. A Markovian structure was used to describe WLLs for individual homes on subsequent rounds of sampling given a set of transitional probabilities, in which homes with higher WLLs at baseline were more likely to exhibit a spike on repeated sampling.

**Results:**

Assuming 2% of homes had a spike on the first round of sampling and a mid-range estimate of transitional probabilities, the initial round of sampling had a 6.4% sensitivity to detect a spike. Seven rounds of sampling would be needed to increase the sensitivity to 50%, which would leave unrecognized the more than 15,000 homes that intermittently exhibit spikes.

**Significance:**

For assessing household risk for lead exposure through drinking water, multiple rounds of water sampling are needed to detect the infrequent but high spikes in WLLs due to particulate release. Water sampling procedures for assessment of lead exposure in individual homes should be modified to account for the infrequent but high spikes in WLL.

**Impact:**

It has been known for decades that intermittent “spikes” in water lead occur due to the sporadic release of lead particulates. However, conventional water sampling strategies do not account for these infrequent but hazardous events. This research suggests that current approaches to sampling tap water for lead testing identify only a small fraction of homes in which particulate spikes occur, and that sampling procedures should be changed substantially to increase the probability of identifying the hazard of particulate lead release into drinking water.

## Introduction

Testing household drinking water for lead is important in efforts to reduce lead exposure in households. Water lead levels (WLLs) can vary among homes within a community due to between-home differences in the presence, type, and condition of lead service lines, and of lead-containing plumbing fixtures in homes [1–3]. Even at the kitchen tap of an individual home, elements of water sampling protocols – such as the flow rate, duration of water stagnation prior to sampling, and the volume that flows from the tap prior to collecting the water sample can impact WLL, as can water temperature, and seasonality [3–6]. Distinct from variability due to these factors, water samples within a given home—collected using the same protocol on different days – can vary substantially. The “…seemingly random, but very high, metal concentrations in water samples” were described more than 30 years ago [7]. Such sporadic “spikes” in WLLs have been described in homes served by community water systems [4, 5, 8, 9], schools [10, 11], and correctional facilities [12, 13], as well as homes served by private wells [14–17]. One recent study defined a transient peak operationally as “…a single sample or proximate samples (e.g., 4th and 6th L) with Pb concentrations above a threshold (≥15 μg/L) and considerably higher (×2 or more) than other measurements at that location [18].” Spikes have been attributed to the sporadic release of lead particles from lead service lines [4, 9], brass plumbing materials in homes [4, 5], solder [17, 19, 20], components of private wells [17], and scale lining the inner surface of pipes to which lead has been complexed [2, 8]. Particulate lead has generally been defined operationally as lead that is captured by a 0.45μm pore filter, though recent work has defined soluble lead as the fraction that passes through a 0.2 μm filter [21] or an ultrafiltration process [8]. When present, particulate lead can account for the vast majority of the total lead found in water samples, especially in samples with high total lead levels [15, 19].

WLLs are associated with blood lead levels (BLLs) in children [22–25] and recent work noted associations between year-to-year increases in WLLs at the community level in Massachusetts with lower math scores among children in those communities [26]. Importantly, particulate lead in water appears to contribute to elevated BLLs. In vitro methods [27] have indicated that lead from plumbing materials is bioavailable [28]. The Integrated Exposure Uptake Biokinetic (IEUBK) model has been used to estimate BLLs among Montreal children based on the evaluation of tap water particulates. That work estimated that among children whose tap water has approximately 20 µg/L of particulate lead, 42% would be expected to have BLLs >5 µg/dL, while children in households where water particulate lead levels exceed approximately 76 µg/L, 90% would have BLLs >5 µg/dL [29]. Thus, identifying lead particulates, even if they are present sporadically, has public health significance.

The US Environmental Protection Agency’s Lead and Copper Rule (LCR) was developed for monitoring WLLs and corrosion control in public water systems [30]. Under the LCR, repeated sampling of a home is required if a water utility adds a new water source or makes a long-term change in treatment processes [31]. Though it was not developed for evaluating lead exposure at the household level, state agencies have recommended the LCR water sampling approach for evaluating lead risk at individual homes: the collection of a “first draw” and, in some cases, a flushed water sample after overnight water stagnation [32–36]. While that approach may be adequate for evaluating soluble lead levels, it does not address the infrequent but high spikes in WLLs due to the sporadic release of particulate lead. Others have noted this limitation and its potential significance for homeowners [37–39]. Masters and colleagues have noted that because of the seemingly random occurrence of particulate lead spikes, “…[e]ven collection of several samples according to this [LCR] criterion can indicate a water is safe, when it is in fact highly hazardous [19].”

It is not known how many sampling events are needed to achieve a specified degree of confidence that WLL spikes do not sporadically occur in a given home or institution. Pipe rig studies have found that despite standardization of flow conditions, WLLs varied substantially, with relative standard of variation in the range of 20–60%, depending on the composition of the pipe materials [6]. Based on that observation, the authors noted that the number of samples for LCR compliance purposes would have to be increased substantially to account for WLL variability. Likewise, a study of WLLs in schools noted comparable variability and concluded that the number of samples collected for monitoring would have to be increased 10-fold or more to accurately estimate mean WLLs [11]. Longitudinal studies of the frequency, magnitude, and predictors of spike occurrence are lacking. As a result, it is not known how many times drinking water should be sampled and analyzed to achieve a specified level of confidence that water lead spikes are unlikely to occur in a home. The aim of this study is to address that knowledge gap.

## Methods

Datasets of WLL test results for individual homes were generated by simulation. Each dataset consisted of WLL results for 100,000 hypothetical homes, each sampled once per day for 15 days. The simulation of WLL at individual homes at each round of sampling rested on two specifications: (1) the WLL of each home on the first round of sampling and (2) transitional probabilities, which describe the probability of WLL (non-detect, detect but no spike, spike) in a subsequent home water sample, given the WLL in the preceding sample.

### Specification 1: initial WLL results for each home

We used observational data as the basis for the distribution of water WLLs in the initial round of sampling in each set of 100,000 homes. The first type of observational data was WLLs collected to meet requirements of the LCR from one large Midwestern state, Illinois, and one large Eastern state Pennsylvania, for water samples collected between January 1, 2020-June 30, 2021 (both datasets are available at 10.5061/dryad.gb5mkkwtv). These data represent WLLs collected in communities by public water systems. We limited LCR data analyses to samples collected from homes by excluding data from samples collected upstream of water distribution systems, in distribution systems, as well as “entry point” samples (before flowing into a home). As a simplification, we categorized the observed WLL values (measured on a continuous scale of µg/L) into three categories: non-detect, detectable but below the 15 µg/L LCR action level, and ≥15 µg/L. The rationale for this categorization is the assumption that homes with non-detectable WLL are least likely to periodically exhibit spikes due to the sporadic release of particulate lead, while homes with WLLs that are detectable but below the action level are more likely to exhibit spikes, and that homes with WLLs exceeding the action level are more likely still to exhibit WLL spikes.

The second type of observational data used in specifying the distribution of WLLs in homes on initial testing comes from published community-based studies of WLL. We excluded studies of outbreak investigations or targeted sampling of communities known to have very high WLLs. Findings of those studies are summarized in Table 1.

**Table 1:** WLL in first draw samples in studies with defined sampling frames and at least 100 homes/buildings.

Setting	% of samples ≥10 µg/L	% of samples ≥15 µg/L
273 Chicago homes before water main replacement [18]	4.0%	0.4%
151 Illinois homes with private wells [16]		3.3%
3868 Wisconsin homes with private wells [45]		1.8%
2144 Virginia homes with private wells [14]		19%
251 Pennsylvania homes with private wells [46]		12%
31,679 samples from 8350 non-residential buildings in 4 Canadian provinces [10]	11%	
National survey of 678 US homes [47]	1.2%	0.4%
Washington DC EPA LCR compliance data in the years before and after the period of increased WLLs [48]		~5–10%

*WLL* Water lead level.

The LCR dataset consists of measures of total WLL, without differentiating between soluble and particulate lead. Within the LCR data proportions of WLL attributable particulate lead or lead spikes is not known. For that reason, we assumed that the occurrence of a particulate lead spike was less than the occurrence of a WLL ≥ 15 µg/L and we produced two sets of simulations: a “lower prevalence specification” with 2% of homes having a spike on the initial round of sampling, and a “higher prevalence specification” in with 10% of homes are found to have spike on the initial round of sampling. The 2% occurrence of spikes on the initial round of sampling is well below observed prevalence of WLL ≥ 15 µg/L in the LCR data (Table 2) and in most of research studies of WLL prevalence (Table 1). The “higher prevalence scenarios” of 10% of homes having lead spikes on the first round of sampling may be consistent with the prevalence WLL ≥ 15 µg/L in 19% of homes observed at the upper end of the range of in published community-based studies (Table 1).

**Table 2:** Distribution of water lead levels (µg/L) for January 1, 2020–June 20, 2021 in data reported to state environmental agencies under the Lead and Copper Rule.

WLL category	Pennsylvania N (%)	Illinois N (%)
Non-detect	1674 (50.4)	1188 (59.3)
Detectable, <15 µg/L	1427 (42.9)	743 (37.1)
≥15 µg/L	223 (6.7)	74 (3.7)
Total	3,324 (100)	2,005(100)

### Transitional probabilities

The second specification for the WLL simulations describes the distribution of WLL categories on the n + 1 round of sampling, given the results from the n round of sampling. Little empirical information is available to characterize the frequency of spike occurrence, and less still is known about the probability of a spike occurring in a home, given the WLL in a water sample collected previously from the same home. The best available data to estimate the frequency of spike occurrence comes from a study conducted by Batterman and colleagues that involved repeated tap water sampling at Chicago homes before and after water main replacement (not lead service line replacement). In that study, 12 of 273 homes (4.4%) demonstrated a spike, defined as a WLL of ≥15 µg/L in a sample that was at least twice as high as other measurements at that home (two of those homes had spikes of >100 ppb) (Supporting Information Table S3A of Batterman) [18]. We assumed that the occurrence of a spike on a given round of sampling would be less likely—though possible—in homes with undetectable lead on the prior round of sampling, and more likely in a home that on the prior round of sampling had detectable lead. The “mid-range” transitional probabilities, summarized in Table 3 are informed by the findings of Batterman et al.: the 0.05 probability of a spike following a detectable WLL is based on the Batterman study’s 4.4% observed frequency of spike occurrence. Given the dearth of observational data upon which to base transitional probabilities, we simulated WLL in homes using the three sets of probabilities, each with a different “probability of upward transition,” which refers to the likelihood of the subsequent water sample (round n + 1) being in a higher WLL category than in the present round of sampling (round n). For example, in the “higher probability of upper transition” set of specifications, a WLL that was detectable but <15 μg/L on round n, would have a 0.05 probability (5%) of having a spike in round n + 1. If a home had a WLL of ≥15 µg/L on round n, the probability of having no detectable lead in round n + 1 would be 0.15, the probability of a detectable lead but no spike would be 0.80, while the probability of spike would be 0.05.

**Table 3:** Transitional probabilities for repeated water sampling rounds, based on water lead categories of the prior sample.

Scenario	WLL category on round_n_	Probability of WLL categories on Round_n+1_			
		Non-detect	Detectable, no spike	Spike	Total
Higher probability of upward transition	Non-detect	0.75	0.20	0.05	1.0
	Detect but <15 µg/L	0.10	0.65	0.25	1.0
	≥15 µg/L	0.05	0.70	0.25	1.0
Mid-range probability of upward transition	Non-detect	0.80	0.19	0.01	1.0
	Detect but <15 µg/L	0.30	0.65	0.05	1.0
	≥15 µg/L	0.15	0.80	0.05	1.0
Lower probability of upward transition	Non-detect	0.90	0.095	0.005	1.0
	Detect but <15 µg/L	0.30	0.69	0.01	1.0
	≥15 µg/L	0.15	0.825	0.025	1.0

The primary set of model specifications for creating a dataset of WLLs for 100,000 homes tested 15 times were, (1) a 2% prevalence of spikes on initial testing, and (2) the mid-range probabilities noted in Table 3. These assumptions were varied to generate a broader range of potential scenarios using the alternative transitional probabilities in Table 3, as well as the higher prevalence of spike occurrence at baseline.

Simulation: The simulation was conducted using R (10.5061/dryad.gb5mkkwtv). For each scenario a series of WLL results were generated for each of 100,000 homes and for baseline plus 14 additional rounds of testing using the transitional probabilities (Table 3). A Markovian structure was used in which each daily measurement depends on the previous one via the transitional probabilities. The Markovian paradigm suggests that tomorrow’s WLL category depends on today’s WLL category, and today’s WLL Category depends on yesterday’s. However, tomorrow’s WLL category does not directly depend on yesterday. Rather, tomorrow’s WLL Category depends on yesterday’s only through today’s WLL category. The choices of initial and transitional probabilities, along with the repeated measures design, results conceptually in a 15-dimensional hypercube where available information is stored at its vertices. The relative positions of these vertices are coded into a design matrix where patterns that are similar in a metric (in terms of WLL Categories and/or rounds) tend to be more highly correlated than patterns that are far apart. The data generation procedure for WLL results for each home in each round of sampling following the initial round is:

1 $$P\left( {X_{i,j} = k|X_{i,j - 1} = m} \right)$$

where *i* denotes homes and ranges from 1 to 100,000 and *j* denotes measurement round 2 through 15. If *j* is today, then *j*-1 was yesterday; *k* and *m* are the WLL Category in which 0=undetectable lead, 1=detectable but not a spike, 2=spike. For example, P(X_59,8_ = 2 | X_59,7_ = 1) means the probability that the WLL Category for home 59 on round 8 of sampling will exhibit a spike, given that the same home had a detectable WLL < 15 µg/L in round 7.

Analyses of the simulated datasets was conducted using SAS version 9.4 (SAS institute, Cary NC).

The sensitivity of detecting a particulate lead spike in *j* rounds of sampling was calculated as the percent of homes with lead detection in any round through round *j* relative to the number of homes in which a particulate lead spike was detected in any round through *j* = 15. For the homes that have undetectable lead on initial sampling, we summarized the probability of identifying a spike in WLL on any subsequent rounds of sampling. This process was repeated for homes that have no detectable lead in the first two, the first three, the first four, etc., rounds of sampling.

## Results

The frequency of WLL spikes observed by each round of sampling, assuming a 2% prevalence of spikes in the initial round of sampling and the mid-range set of transitional probabilities is presented in Table 4. A total of 31,720 (31.72%) of the 100,000 homes were found to have a WLL spike on one or more of 15 rounds of sampling. Thus, if only one round of sampling had been conducted, only 2,021 of 31,720 homes with spikes (6.4%) would have been identified while 29,699 of homes that exhibit a spike on any of 15 rounds of sampling would have not have been identified. A 50% sensitivity to spike detection would only be achieved after seven rounds of sampling, and at that point 15,178 homes that do exhibit spikes in any of 15 rounds would remain undetected.

**Table 4:** Results of water sampling and lead analysis from 100,000 homes, and sensitivity to detecting water lead spikes.

Round	Number (%) of homes with first spike occurrence	Cumulative Frequency (%) of homes with at least 1 spike	Homes with undetected spikes	Cumulative sensitivity
1	2021 (2.02)	2021 (2.02)	29,699	6.37%
2	2949 (2.95)	4970 (4.97)	26,750	15.67%
3	2589 (2.59)	7559 (7.56)	24,161	23.83%
4	2353 (2.35)	9912 (9.91)	21,808	31.25%
5	2299 (2.3)	12,211 (12.21)	19,509	38.50%
6	2183 (2.18)	14,394 (14.39)	17,326	45.38%
7	2147 (2.15)	16,541 (16.54)	15,178	52.15%
8	2075 (2.08)	18,616 (18.62)	13,104	58.69%
9	1959 (1.96)	20,575 (20.57)	11,145	64.86%
10	2049 (2.05)	22,624 (22.62)	9096	71.32%
11	1912 (1.91)	24,536 (24.54)	7184	77.35%
12	1844 (1.84)	26,380 (26.38)	5340	83.17%
13	1857 (1.86)	28,237 (28.24)	3483	89.02%
14	1762 (1.76)	29,999 (30.00)	1721	94.57%
15	1721 (1.72)	31,720 (31.72)	0	100.00%

Simulation: lower baseline prevalence and more conservative set of transitional probabilities.

The impacts of varying the assumptions regarding baseline prevalence and the transitional probabilities on the number of homes found to exhibit WLL spikes, and the sensitivity of a single round of sampling to identify homes at risk for spikes is summarized in Table 5. This demonstrates that, given the simulation specifications, between 5 and 7 rounds of sampling would be needed to achieve of 50% sensitivity for spike detection.

**Table 5:** Distribution of baseline (Round 1) water lead categories and spike detection on any of 15 rounds.

Prevalence of spike on Round 1 of sampling	Probability of upward transition	Spike detection on any round	Initial round of sampling sensitivity to spike detection	50% sensitivity achieved by
2%	Lower	10.51%	19.23%	Round 6
2%	Mid-range	31.72%	6.37%	Round 7
2%	Higher	89.74	2.25%	Round 5
10%	Mid-range	38.51%	25.89%	Round 5

**Fig. 1:**
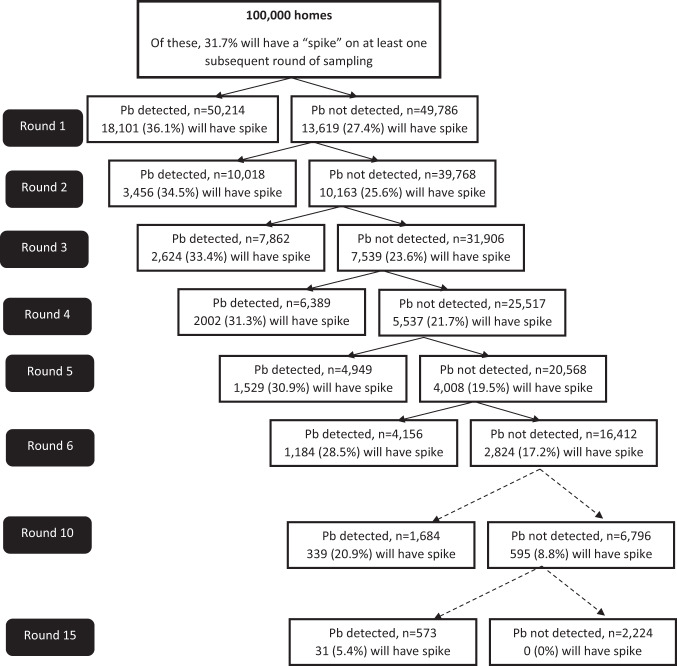
Repeated sampling of homes with consecutive lead non-detection. Results of repeated testing of 100,000 homes with undetectable lead on initial sampling, in the 2% prevalence, mid-range probability of upward transition.

We evaluated whether a series of consecutive non-detects of lead at an individual homes can provide reassurance that a home is unlikely to exhibit lead spikes. As summarized in Fig. 1, with each consecutive non-detect, the probability of spike occurrence in any of the subsequent rounds of sampling decreases. Under the 2% baseline prevalence, mid-range set of transitional probabilities, the first five consecutive rounds of water sampling will all test negative for lead in 20,568 homes. However, water samples from 4008 (19.5%) of those homes had lead spike in a subsequent round of sampling. Even after 10 consecutive non-detects, 8.8% of homes would have a spike by round 15.

## Discussion

If WLLs in a given home never varied, a single sample would be sufficient to characterize lead levels (assuming no measurement error). However, prior studies have noted that the intermittent release of lead particulates from plumbing or well components renders the collection of single water samples of limited value in characterizing the health risks faced by building occupants [1, 2, 11, 19]. We found that in the scenario of 2% of homes having a spike on the first round of water sampling and the mid-range set of transitional probabilities, approximately 50% of homes have undetectable lead levels, 48% have detectable levels without a spike caused by particulate release, yet if all homes underwent 15 rounds of sampling, spikes of particulate lead release would be identified in 31.7% of homes (Table 5). Thus, only 6.4% of the homes in which intermittent lead spikes occur would be detected on the initial round of sampling, a very low sensitivity to this children’s health hazard. In a community in which 10% of homes have WLL of 15 µg/L or greater (the higher prevalence scenario, consistent with a community in which the public water system exceeds the LCR action level for lead), the sensitivity of a single round of water sampling and analysis was higher (25.9%), but the identification of very high but infrequent particulate lead spikes would be missing in nearly three quarters of all homes in which such spikes occur. We also note that homes with repeated “non-detects” remain at risk for particulate release. Our estimation that 5–8 rounds of sampling would be needed to attain 50% sensitivity to spike detection is consistent with the conclusions of prior studies, such a pipe rig experiment that noted “a very high number of samples would have to be collected under a range of flow conditions” to account for the inherent variability in WLLs due to particulate release [6]. Likewise, and an analysis of WLL variability in samples from schools estimated that a 10-fold increase in sampling may be needed to account for sporadically high WLLs [11].

Though extremely high lead levels can occur sporadically in home drinking water, some state public health agencies and water utilities recommend one round of water sampling, typically after overnight water stagnation (the LCR sampling approach) [32–36]. These guidelines recommend collecting two 1 L samples (generally the first, and the 5th or 6th liter for the purpose of differentiating premise plumbing vs. the service line as the lead source), and as a result, they do not account for the infrequent release of lead particulates into tap water. As summarized in Table 5, five to seven rounds of sampling would be needed to reach a 50% sensitivity for spike identification.

The immediate public health danger of failing to recognize the true level of lead hazard in households is that children’s exposure would continue without interventions. Another impact of failure to capture the short-term variability in household water samples due to spike occurrence is the underestimation of the strength of association between WLLs and blood lead levels (BLLs). Studies of association between WLL and BLL in children have found no association [40] or statistically significant but relatively weak associations between WLL and BLL in children, with WLL accounting for less than 5% of the variability in BLL, and/or with wide confidence intervals around measures of association [22–25]. Others have observed that the associations – while real and concerning—would likely be stronger if WLLs were not impacted by water temperature, pH and other sources of variability and if tap water consumptions were better characterized [22, 41]. Mischaracterizing the water lead hazard at the household level due to sporadic particulate lead release would weaken associations between WLL and BLL in children, as well as recently reported associations between WLL and scores on standardized math exams in children [26]. Some environmental and/or public health authorities advise repeated testing if high WLLs are measured [36, 42]. If the infrequent intermittent release of particulate caused those initial high levels, it would be unlikely to randomly occur on the follow-up round of sampling release of lead particulates. This would falsely indicate the resolution of hazard to children’s health. We found that, given the simulation inputs, extensive sampling efforts are needed to establish with confidence that spike occurrence is unlikely in a given home.

From a policy standpoint, one might wonder whether such extensive sampling is justified, and perhaps household level lead inspections of plumbing and private wells may result more directly in lead remediation. Repeated sampling and analysis would be costly, especially for homeowners with private wells who would not be able to work with community water systems to identify drinking water lead (costs for lead sampling kits, shipping, and sample analysis are in the range of $40–$125). Prioritizing homes with detectable water lead on initial testing, older homes, and those in communities in which particulate lead has been noted to occur may be a reasonable response to an initial round of sampling, but it should not be the end of lead assessments in the community. As summarized by Pieper and colleagues, homes with private wells are not subject to the Federal requirement of using “lead free” materials [17]. Additionally, only public water systems are required to test for lead under the LCR, with very few state-level requirements for private well-owners to test their drinking water for lead [14, 17]. Given the reports of high WLLs and particulate lead presence in studies of homes with private wells [15–17], efforts to detect WLLs should be extended to rural areas and other settings where private wells are common.

Other approaches to identifying particulate release have been described. Clark and colleagues demonstrated that water samples from some homes had higher levels of particulate lead at flow rates estimated to be 3–10 L/min or at rates estimated to be 4–14 L/min than when the same tap was sampled using low flow rates (estimated to be 1 L/min) [4]. Likewise, water sampling at drinking water treatment plants through pipe rigs found that with flow rates of 10 L/min, particulate lead concentrations were sometimes 100-fold greater and far more variable than when flow rates were 2 L/min [19]. Thus, high flow sampling should be able to increase the sensitivity of individual household water samples for particulate lead. A complementary approach to identifying homes in which intermittent lead particulate release occurs would be the collection of large volumes of water on a single occasion (rather than 1 L/day over 15 days in the present simulation), using filters that capture lead particulates and then quantifying the mass and lead content of particles trapped on the filter material [8]. Because disturbances in the area of the home, such as street excavation or work on water mains is associated with higher WLLs [43], testing on multiple days may increase the likelihood of identifying high WLLs. Future research would be needed to determine whether filtrate from a single 15-L sample collected under high-flow conditions provides comparable information to 1-L samples collected using conventional flow rates on 15 days. Likewise, the optimal temporal spacing of repeated sampling (once per day, once per week, once per month, etc) is not known.

The findings of this research are subject to several limitations. The simulation of spike occurrence relied on a set of transitional probabilities. We are aware of only one field study that systematically sampled homes repeatedly and reported spike occurrence [18] and we used information from that study to develop the mid-range transitional probabilities. However, the focus of that study was not spike occurrence and the details of WLLs of the sample prior to a spike were not reported. Thus, the transitional probabilities that we utilized in the simulations could be too high or too low. Furthermore, the occurrence of spikes may vary by age of public water systems as well as age of homes in communities (e.g., those built before vs. after the implementation of amendments to the Safe Drinking Water Act that reduced the lead content of plumbing materials) [44]. Additionally, the corrosivity of water would likely impact the transitional probabilities, given the association between corrosivity and lead release from service lines and plumbing materials. Thus, further work is needed to refine and add confidence bounds around the transitional probabilities based on repeated sampling of water (as opposed to profile sampling, which is designed to help identify lead sources within a home-service line system). As more becomes known about transitional probabilities in various “real world” settings, assumptions used in the present study would likely require revision. It is possible that the assumptions we made regarding baseline WLL prevalence and transitional probabilities are not conservative enough, as they were not based on WLL collection methods that are likely to promote particulate release, namely, high-flow sampling [4, 19]. The water lead categories used in the simulation are a simplification, considering non-detection, soluble lead, and particulate spikes only. In reality, multiple dynamic factors are at play. These include within-home factors, such changes in flow, temperature, corrosivity and physicochemical fluctuations in water characteristics, as well as differences between homes in the composition of plumbing materials. As a result, it is possible that WLL at concentrations below 15 µg/L can be predominantly in particulate form and increases in WLL to greater than 15 µg/L can be due to higher concentrations of soluble lead.

We conclude that insufficient water sampling of tap water from homes likely results in failure to identify infrequent but, from a health standpoint, concerning spikes in water lead content. The process we used for simulating WLLs may be useful in developing sampling strategies for other environmental hazards that exhibit seemingly random but very high levels infrequently. Further research is needed to fine-tune the specific assumptions of the simulation, such as the frequency of particulate lead release as a function of baseline WLL. Finally, research is needed to determine whether large tap water samples (perhaps 15 L) may provide information about water lead spikes that is comparable to that which may be obtained by collecting 1 L samples on 15 occasions. Ultimately, the solution to the problem of childhood lead exposure through drinking water will require the replacement of lead-containing plumbing fixtures, solder, pipes and service lines.

## Data Availability

Statistical code used to generate the simulation datasets, the datasets, and the Lead and Copper Rule data (Illinois and Pennsylvania) are available at 10.5061/dryad.gb5mkkwtv.
